# Weak Coordinating Character of Organosulfonates in Oriented Silica Films: An Efficient Approach for Immobilizing Cationic Metal-Transition Complexes

**DOI:** 10.3390/molecules27175444

**Published:** 2022-08-25

**Authors:** Samuel Ahoulou, Clara Richart, Cédric Carteret, Sébastien Pillet, Neus Vilà, Alain Walcarius

**Affiliations:** 1 LCPME, CNRS, Universite de Lorraine, F-54000 Nancy, France; 2 CRM2, CNRS, Universite de Lorraine, F-54000 Nancy, France

**Keywords:** mesoporous silica films, polypyridyl metal complexes, confinement effects, metal ligand coordination, organic–inorganic hybrids, iron-bipyridine derivatives, modified electrodes

## Abstract

Iron (II) tris(2,2′-bipyridine) complexes, [Fe(bpy)_3_]^2+^, have been synthesized and immobilized in organosulfonate-functionalized nanostructured silica thin films taking advantage of the stabilization of [Fe(H_2_O)_6_]^2+^ species by hydrogen bonds to the anionic sulfonate moieties grafted to the silica nanopores. In a first step, thiol-based silica films have been electrochemically generated on indium tin oxide (ITO) substrates by co-condensation of 3-mercaptopropyltrimethoxysilane (MPTMS) and tetraethoxysilane (TEOS). Secondly, the thiol function has been modified to sulfonate by chemical oxidation using hydrogen peroxide in acidic medium as an oxidizing agent. The immobilization of [Fe(bpy)_3_]^2+^ complexes has been performed in situ in two consecutive steps: (i) impregnation of the sulfonate functionalized silica films in an aqueous solution of iron (II) sulfate heptahydrate; (ii) dipping of the iron-containing mesostructures in a solution of bipyridine ligands in acetonitrile. The in situ formation of the [Fe(bpy)_3_]^2+^ complex is evidenced by its characteristic optical absorption spectrum, and elemental composition analysis using X-ray photoelectron spectroscopy. The measured optical and electrochemical properties of immobilized [Fe(bpy)_3_]^2+^ complexes are not altered by confinement in the nanostructured silica thin film.

## 1. Introduction

The ability of organosulfonate anions to coordinate transition metal centers remains quite unexplored since they are considered as poor ligands not able to displace solvent molecules from the first coordination sphere of a metal ion efficiently [1]. Examples with direct sulfonate-to-metal coordination are rarely described; they are often limited to representative elements or closed shell d^10^ cations (Ag(I) and Cd(II)) [2]. Even though, some examples of frameworks made of divalent transition metal imidazole complexes bearing 1,5-naphtalatedisulfonate have been already reported. They provide direct sulfonate–metal linkages constituting the first known compounds with open d-shell transition metal with octahedral coordination [3]. Weak interactions with metal ions that lead to the formation of robust solids assisted by hydrogen bonding have been also reported in the case of divalent and trivalent metals from the first-row transition metal [4]. In this sense, the ability of sulfonate moieties to interact with cationic species was first observed in the case of guanidium cations that can donate protons for hydrogen bonding leading to the formation of self-assembled “soft” or “flexible” materials. In the case of such soft frameworks based on the guanidium disulfonate system, each sulfonate function possesses three oxygen atoms and six lone pairs that can form six charge-assisted and directional hydrogen bonds with six protons of the flat guanidium cation [5,6,7]. The relatively weak coordination ability of organosulfonates functions to transition-metal centers makes difficult the displacement of ligands such as water and facilitates such hydrogen bonding interactions between the guanidium cations and later on cationic metal complexes [8,9,10]. In contrast with other anionic functionalities such as organic phosphonates and/or carboxylates that can replace weak ligands, the sulfonates tend to hydrogen-bond to the available proton-donating ligands instead. Several layered frameworks have been described with cobalt-based complexes and various disulfonate anions [11,12,13,14].

Nanostructured silica films hosting metal-based transition complexes are interesting for applications in catalysis, for the preparation of optoelectronic devices, or for optical and electrochemical applications [15,16,17,18]. As recently demonstrated, the versatility of the combination of the electrochemically assisted self-assembly (EASA) route to mesoporous silica films [19,20,21] and click chemistry applied to silica materials [22,23] can be exploited to accommodate polypyridyl metal-based transition metal complexes (e.g. [Fe(tpy)_2_]^2+^ [24] and [Fe(bpy)_3_]^2+^ [25]) in such nanostructured materials. In both cases, the immobilization required several steps: (i) generation of azide-functionalized vertically oriented silica films by EASA using tetraethoxysilane (TEOS) and 3-azidopropyltriethoxysilane (AzPTES) as silane precursors; (ii) grafting of the polypyridyl ligands by click coupling with a specifically prepared ethynyl polypyridyl derivative; (iii) coordination of Fe^2+^ ions to the polypyridyl-functionalized silica films by dipping the films in an aqueous solution containing FeSO_4_●H_2_O; (iv) the coordination sphere of the iron (II) was completed by dipping the films in a solution containing bipyridine or terpyridine ligands in acetonitrile to form the final transition-metal coordination complexes [24,25].

The aim of the present work is to propose an alternative to the above time-consuming covalent approach for the immobilization of transition-metal coordination complexes in vertically oriented silica films, based on the use of sulfonate-functionalized silica films as weak coordinating membranes allowing the immobilization of Fe^2+^ centers in a first step. Two distinct hypotheses should be considered to explain the immobilization mechanism of Fe^2+^ centers in the first stage of the modification process: (i) the organosulfonate moieties could act as a second coordination sphere interacting via the formation of hydrogen bonds with the aquo ligands of the [Fe(H_2_O)_6_]^2+^ complex; (ii) direct weak coordination of the organosulfonate functions to the Fe^2+^ centers to form the [Fe(SO_3_-R)_2_(H_2_O)_4_]^2+^ moieties. In both cases, such interactions are obviously weaker than a covalent bond but are strong enough to avoid the spontaneous leakage of the coordination complexes immobilized in the first step of the functionalization process. Then, we demonstrate the ability of organosulfonate to act as a weak coordinating ligand to bind iron (II) cations that can subsequently react with bipyridine ligands to form and immobilize polypyridine complexes in situ in such nanoporous matrices. We finally explore the optical and electrochemical properties of the synthesized [Fe(bpy)_3_]^2+^SO_3_^−^@SiO_2_ films. This work opens the door to the application of such a modification strategy for the immobilization of other species that cannot be easily introduced into the silica matrix by the covalent route.

## 2. Results and Discussion

### 2.1. Generation of Mesoporous Films and Monitoring of the Successive Functionalization Steps

The internal surface of the oriented nanochannels of the mesoporous silica films is negatively charged (due to the presence of silanolate groups at pH higher than the isoelectric point of silica, i.e., 2) [21,26,27]. Therefore, it could in principle be exploited for binding positively charged complexes as [Fe(bpy)_3_]^2+^ via favorable electrostatic interactions. This could be an alternative strategy to the covalent immobilization of transition metal-based cationic complexes in mesoporous silica films that we used previously for confining [Fe(bpy)_3_]^2+^ and [Fe(terpy)_2_]^2+^ in similar mesoporous silica films. However, all attempts to implement this electrostatic immobilization approach to non-functionalized vertically aligned mesoporous silica films prepared by EASA have been unsuccessful in spite of mesopore size (i.e., 2 nm in diameter) larger than the probe size (i.e., 0.8 nm for [Fe(bpy)_3_]^2+^). Cyclic voltammetry experiments (which are commonly used to point out accumulation effects in such films [28]) have not shown any evidence of the presence of those electroactive species accumulated in the nanoporous matrices (see Figure S1) suggesting that their effective immobilization would require significantly stronger interactions than the “simple” electrostatic ones with the negatively charged silanolate groups.

**Figure 1:**
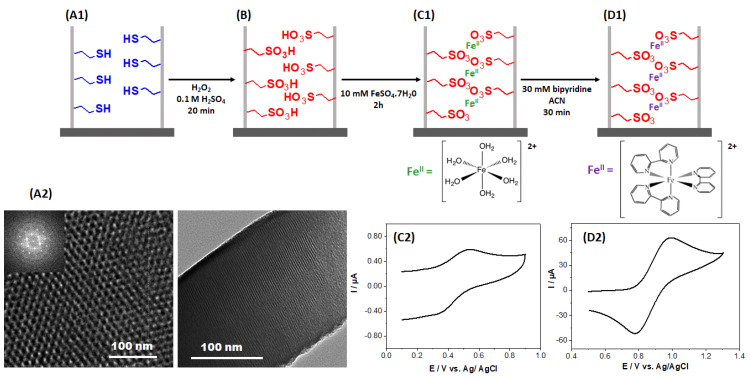
(**A**) Mercaptopropyl-functionalized silica thin films electrochemically obtained by EASA from TEOS and MPTMS as silane precursors ((**A1**): schematic illustration of one mesopore channel; (**A2**): TEM top and cross-section views). (**B**) Chemical oxidation of the mercaptopropyl functions into propylsulfonate using a solution of hydrogen peroxide in 0.1 M H_2_SO_4_. (**C1**) Immobilization of [Fe(H_2_O)_6_]^2+^ in sulfonated-functionalized silica films by immersion in an aqueous solution containing 10 mM FeSO_4_●7H_2_O for 2 h in an inert atmosphere ((**C1**): illustrative scheme; (**C2**): cyclic voltammogram recorded at a scan rate of 50 mV s^−1^ in 0.1 M NaNO_3_ aqueous solution). (**D**) Replacement of the aquo ligands by immersion in an acetonitrile solution containing 30 mM bipyridine ((**D1**): illustrative scheme; (**D2**): cyclic voltammogram recorded at a scan rate of 50 mV s^−1^ in 0.1 M NaNO_3_ aqueous solution).

Based on these observations, a weak coordinating group, i.e., propylsulfonate, has been employed to firstly immobilize Fe^2+^ ions in the nanopores of the oriented silica thin films. To this aim, the vertically oriented silica thin films containing mercaptopropyl functions (Figure 1A1) have been electrochemically generated on ITO electrodes by adapting the previously reported EASA procedure [20,29], using this time TEOS and MPTMS as silane and organosilane precursors. It has been previously demonstrated that a high level of ordering can be obtained for vertically oriented mercaptopropyl-functionalized films prepared by EASA, with hexagonally packed and oriented mesochannels maintained intact in the nanostructure up to 30% MPTMS in the starting sol [30], which is also the case here (Figure 1A2). Then, these grafted thiol functions have been chemically oxidized by dipping the mercaptopropyl-functionalized films in an acidic solution of hydrogen peroxide (Figure 1B). This medium is indeed known to enable the effective oxidation of thiol groups into sulfonate moieties, even in a confined environment as that of silica mesochannels, as confirmed by Raman spectroscopy and XPS [31,32,33]. The sulfonate functionalized films were subsequently dipped in a solution containing 10 mM FeSO_4_●7H_2_O, a medium where all the iron (II) sulfate species dissolved in water to form the corresponding aquo complex, [Fe(H_2_O)_6_]^2+^ (with octahedral molecular geometry), likely to interact with sulfonate moieties (Figure 1C1). Cyclic voltammetry and XPS analysis were employed to prove the presence of iron (II) inside the mesoporous channels. The electrochemical response obtained is shown in Figure 1C2 exhibiting oxidation and reduction peaks around 0.5 V vs. Ag/AgCl assigned to the [Fe(H_2_O)_6_]^2+/3+^ redox system in the silica matrix (XPS analysis will be discussed hereafter). The last step of the functionalization process consisted of the displacement of the aquo ligands by bipyridine molecules completing the coordination sphere of the transition metal (this was achieved by dipping the modified silica film in a 30 mM solution of bipyridine ligands in acetonitrile) to form [Fe(bpy)_3_]^2+^ interacting with sulfonate moieties (Figure 1D1). Figure 1D2 shows the oxidation/reduction signals characteristic of the resulting [Fe(bpy)_3_]^2+^SO_3_^−^@SiO_2_, appearing at more positive potentials due to the change of coordination sphere of the iron center, in good agreement with the electrochemical behavior previously described for [Fe(bpy)_3_]^2+^ covalently attached to the silica walls [25].

**Figure 2:**
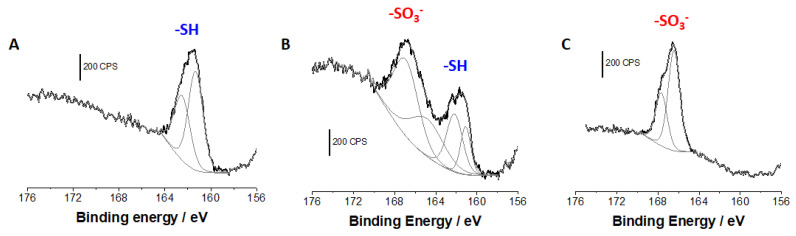
S2p core level spectra of a mercaptopropyl-functionalized mesoporous silica film (obtained by EASA from a sol prepared from 20/80 MPTMS:TEOS), respectively, recorded before (**A**), after 10 min (**B**), and after 30 min (**C**) of chemical oxidation with hydrogen peroxide (30%) in 0.1 M H_2_SO_4_.

XPS analysis of the functionalized mesoporous silica films was performed in order to follow the successive functionalization steps and quantify the film composition at each of these (see Figure S2). As an example, the XPS survey spectrum obtained for a 20/80 mercaptopropyl-functionalized silica film is given in supporting information. It shows the C1s (285 eV), O1s (532 eV), S2s (230 eV), S2p (162 eV) and Si2p (100 eV) characteristic peaks (Figure S2d). The S2s and S2p core level spectrum can be used to monitor the chemical oxidation of the mercaptopropyl functions inside the nanopores. Figure 2 shows the evolution of the S2p core level spectrum before (Figure 2A) and after 10 and 30 min of chemical oxidation using hydrogen peroxide (Figure 2B,C, respectively). In Figure 2A, the S2p core level spectrum shows an asymmetric peak at 161.6 ± 0.2 eV assigned to the thiol groups [30]. The presence of such not very well-resolved doublet is attributed to the low sulfur content in the sample [34]. As is expected, the amount of reduced sulfur (thiol, -SH) decreases progressively during chemical oxidation with hydrogen peroxide, and a new S2p peak appears centered at 167 eV attributed to the oxidized form of sulfur into sulfonate, SO_3_^−^. After 10 min of chemical oxidation, about 50% of the initial thiol functions are transformed into sulfonate (Figure 2B) whereas after 30 min the oxidation is quantitative, and all the sulfur is in the form of sulfonate (Figure 2C).

**Figure 3:**
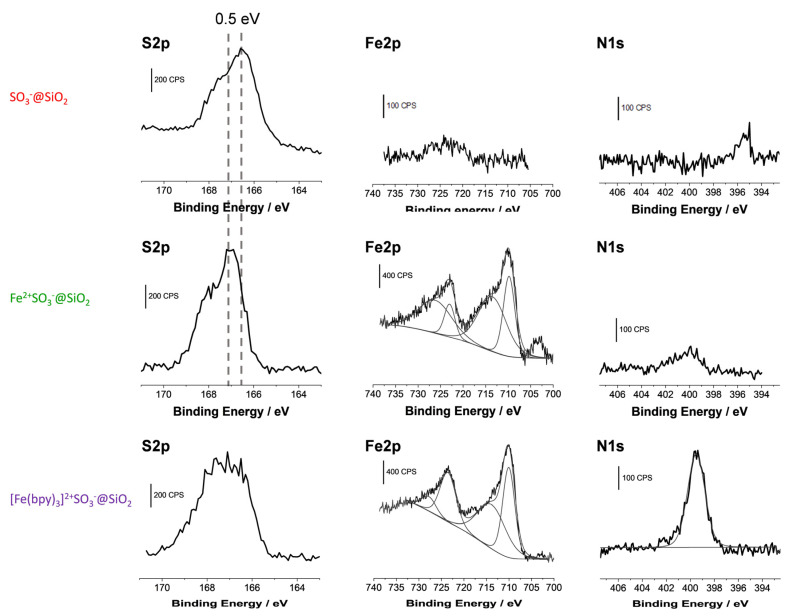
S2p (left), Fe2p (center) and N1s (right) core level spectra recorded for 20/80 films, respectively, functionalized with sulfonate groups (SO_3_^−^@SiO_2_, top), after complexation with iron (II) (Fe^2+^SO_3_^−^@SiO_2_, middle) and after Fe^2+^ complexation and bipyridine displacement of aquo ligands ([Fe(bpy)_3_]^2+^SO_3_^−^@SiO_2_, bottom).

The next step of the functionalization process consisted in the coordination of Fe^2+^ in the sulfonate-functionalized silica films (Fe^2+^SO_3_^−^@SiO_2_). This was also characterized by XPS to determine the iron loadings as a function of the composition of the films. The survey spectra were recorded on different spots for each modified film and the average values of atomic composition and binding energy are gathered in Table 1. For Fe^2+^SO_3_^−^@SiO_2_ films, Fe2p peaks appeared in addition to C1s (285 eV), O1s (532 eV), S2p (167 eV), and Si2p (100 eV) (Figure S2b), and they grew evidently with the sulfonate content in the material (Table 1). Figure 3 displays the narrow S2p (left), Fe2p (center), and N1s (right) core level spectra obtained with 20/80 films, respectively, for SO_3_^−^@SiO_2_ (top), Fe^2+^SO_3_^−^@SiO_2_ (middle), and [Fe(bpy)_3_]^2+^SO_3_^−^@SiO_2_ (bottom). For Fe^2+^SO_3_^−^@SiO_2_, the main envelope of the Fe2p curve fitted with Fe2p3/2 and Fe2p1/2 peaks centered at 710.7 and 723.5 eV, respectively, corresponding well to the presence of Fe^2+^. On the other hand, the interaction of the iron with the sulfonate moieties leads to a shift of 0.5 eV of the binding energy values corresponding to the peaks observed in the S2p core level spectrum due to a change in the sulfonate environment. The resulting S/Fe atomic ratios were close to 2 (Table 1), suggesting the binding of one Fe^2+^ to two SO_3_^−^, consistent with the charge balance. The last step of the modification process involved the displacement of aqueous ligands by bipyridine resulting in a change in the coordination sphere of the metal center with the in situ formation of the [Fe(bpy)_3_]^2+^. In this last step, the sulfonate stops acting as a weak ligand in the second coordination sphere of the iron and behaves as a counter ion in the [Fe(bpy)_3_]^2+^SO_3_^−^@SiO_2_ material. Furthermore, the presence of bipyridine ligands and the in situ formation of the Fe(bpy)_3_^2+^ is demonstrated by the emerging N1s component at 399.5 eV attributed to the bipyridine ligands, with N/Fe atomic ratios close to 6 (Table 1) in agreement with the stoichiometry of the [Fe(bpy)_3_]^2+^ complex, confirming the success of its formation in the [Fe(bpy)_3_]^2+^SO_3_^−^@SiO_2_ films prepared with concentrations of silica precursors up to 20/80 MPTMS/TEOS. Increasing further the amount of organic groups in the mesopore channels results in a higher degree of occupation of the space available inside the matrix making difficult the replacement of aquo ligands by the bulky bipyridine moieties in the last step of the modification process which is evidenced by the significant decrease in the N/Fe ratio for the 30/70 MCTMS/TEOS films.

### 2.2. Structural Characterization of [Fe(bpy)_3_]^2+^ Complexes Formed in the Films

**Figure 4:**
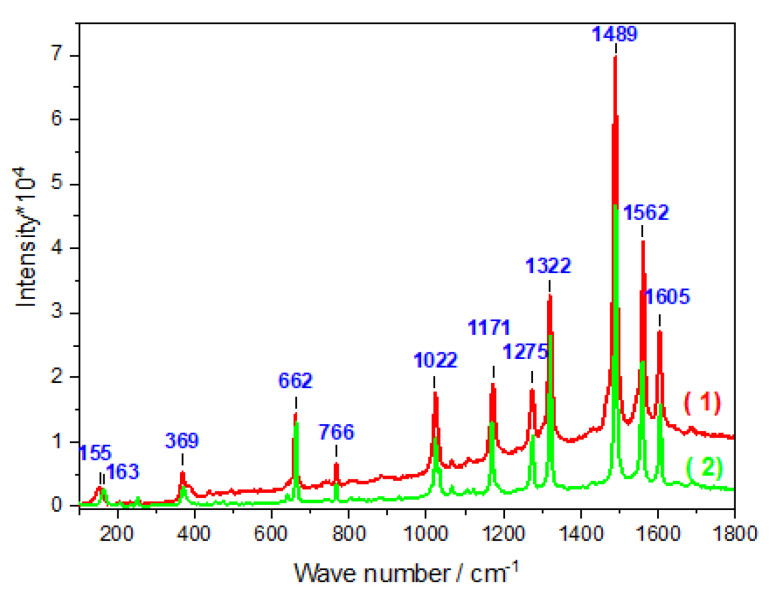
Comparison of the Raman spectra of [Fe(bpy)_3_]^2+^SO_3_^−^@SiO_2_ film (red, 1) and the Raman spectra of [Fe(bpy)_3_]SO_4_●7.5H_2_O microcrystalline powder (green, 2).

Structural characterization of the [Fe(bpy)_3_]^2+^ complex confined in silica films was performed by resonance Raman spectroscopy, measured in the [100–1800] cm^−1^ range as depicted in Figure 4. The spectra have been obtained under resonance conditions with the metal(d) → bpy(*) metal-to-ligand charge transfer (MLCT) absorption band using 532 nm excitation, therefore enhancing vibrational modes which are vibronically active in the corresponding electronic transition. For comparison purposes, and for identification of the exact complexes that were formed in situ in the silica films, we can use the Raman spectrum obtained previously on covalently attached [Fe(bpy)_3_]^2+^ complexes in ordered silica thin films [25] as a benchmark.

The Raman spectrum of [Fe(bpy)_3_]^2+^ and corresponding vibration modes can be separated into three categories: ring modes, inter-ring vibrations (within a bipyridine ligand), and metal–ligand modes. In the 1100–1610 cm^−1^ wavenumber range, the Raman spectrum is strongly dominated by bands arising from the skeletal stretching vibrations of the bpy ligands. This indicates that the electron transfer is extensively delocalized over the aromatic ring system [35]. In particular, the mode at 1489 cm^−1^ is attributed to the C=C stretching mode, the bands at 1275, 1322, and 1562 cm^−1^ correspond to the C-H bending mode [36], while the C=N stretching mode is located at 1605 cm^−1^ This is consistent with the assignment of vibrational bands for [Fe(bpy)_3_]^2+^ and [Ru(bpy)_3_]^2+^ proposed in the literature from resonant Raman, infrared, and DFT calculations [36,37,38,39,40]. The bands observed at 662, 766, and 1022 cm^−1^ are characteristic of ring deformation. The 155 cm^−1^ frequency has been attributed to a combination of chelate-ring modes, benzene cycle bending modes, and N-Fe-N bending modes. The observed band at 369 cm^−1^ corresponds to the Fe-N stretching mode [41]. The complete corresponding band wavenumbers and proposed assignments are summarized in Table 2. Otherwise, the resonance Raman spectra of [Fe(bpy)_3_]SO_4_●7.5H_2_O microcrystalline powder and Fe(bpy)_3_^2+^/SO_3_^−^@SiO_2_ are perfectly overlapped, thus indicating that the structure of the [Fe(bpy)_3_]^2+^ complex is maintained within the silica. Additionally, the complete Raman spectrum of [Fe(bpy)_3_]^2+^, whether it is immobilized in the mesoporous silica thin film through covalent interactions (our previous work [25]) or through hydrogen bonds (the present work), is almost identical with marginal band shift (at maximum 2–3 cm^−1^). This allows to confirm that in both cases, as deeply discussed in [25], the confined complex [Fe(bpy)_3_] is in the formal 2+ oxidation state, and LS electronic state. This is proved by the frequency of the Fe-N stretching mode, observed at 369 cm^−1^. This band would shift to nearly 200–250 cm^−1^ for the Fe(II) in the HS state [41,42,43,44,45].

### 2.3. Electrochemical Response of the [Fe(bpy)_3_]^2+^SO_3_^−^@SiO_2_ Films

**Figure 5:**
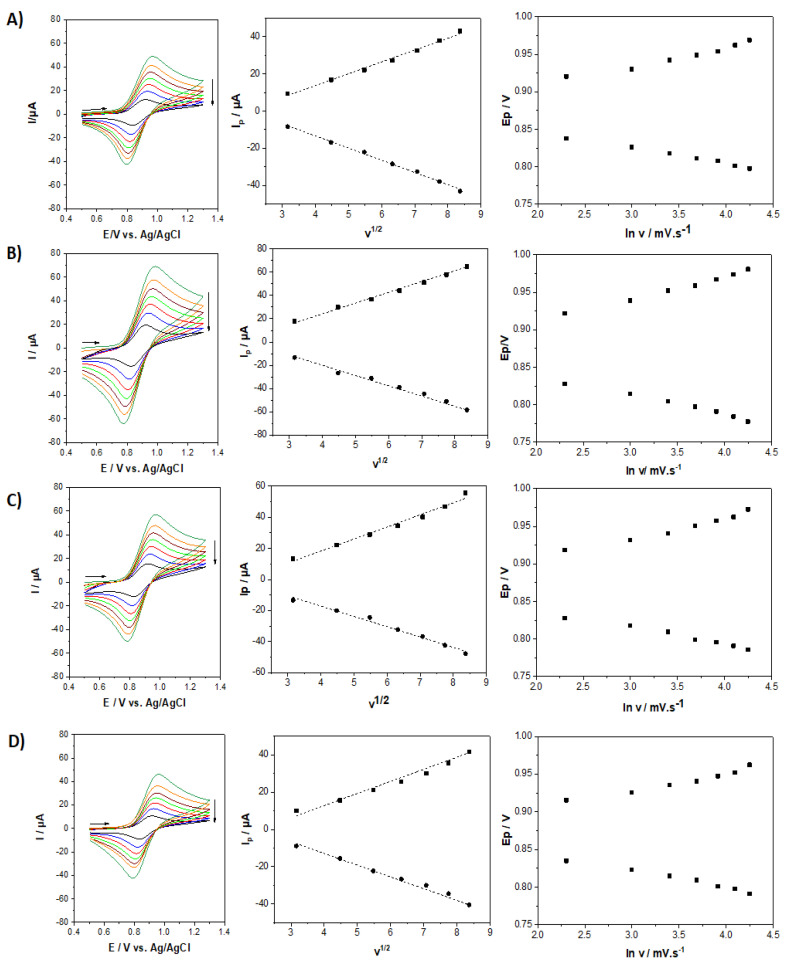
Cyclic voltammograms of [Fe(bpy)_3_]^2+^SO_3_^−^@SiO_2_ functionalized films recorded at potential scan rates of 10, 20, 30, 40, 50, 60, and 70 mV s^−1^ in an aqueous electrolyte solution containing 0.1 M NaNO_3_. Left: CVs of the films prepared from various MPTMS/TEOS ratios (total concentration of silane precursors: 200 mM): 30/70 (**A**); 20/80 (**B**); 10/90 (**C**); 5/95 (**D**). Middle: Dependence of the peak current with the square root of the potential scan rate. Right: Evolution of peak potentials as a function of the logarithm of potential scan rate.

Figure 5 shows the evolution of the electrochemical response obtained by cyclic voltammetry (CV) for four mesoporous silica films bearing [Fe(bpy)_3_]^2+^ at various functionalization degrees (5, 10, 20, and 30% organosilane) recorded in an aqueous 0.1 M NaNO_3_ solution at scan rates varying from 10 to 70 mV s^−1^. The cyclic voltammograms are related to the [Fe(bpy)_3_]^2+/3+^ redox couple centered at 0.87 V vs. Ag/AgCl. CV curves clearly exhibit an evolution related to the functionalization degree. An increase in the anodic and cathodic peak current values is observed with increasing the level of functionalization from 5% up to 20% of sulfonate groups (see left of Figure 5, from D to B), as a result of increasing amounts of [Fe(bpy)_3_]^2+^ in the film. As a matter of fact, a higher concentration of sulfonate groups results in a higher amount of immobilized [Fe(bpy)_3_]^2+^ species, and therefore a more intense electrochemical response. Above this 20% value (i.e., highlighted for 30% of sulfonate groups in the film), the peak currents dropped significantly (Figure 5A). One plausible explanation for this observation is the lack of free space for [Fe(bpy)_3_]^2+^ to form in the mesoporous channels due to steric hindrances when larger amounts of organosulfonate groups are initially present in the starting film, leading to the formation of a lower amount of [Fe(bpy)_3_]^2+^ units under these conditions. The optimal situation corresponding to the largest currents is thus that corresponding to the film prepared using a 20/80 MPTMS/TEOS ratio. For this film, a charge of 236 µC can be extracted from the integration of the anodic signal, corresponding to 18.6×10^−10^ mol [Fe(bpy)_3_]^2+^. It is noteworthy that CVs were stable upon multiple successive potential scans (yet with a small intensity decrease in the first 10–15 cycles, which might be due to some loss of loosely bonded [Fe(bpy)_3_]^2+^ species, but after that the CV curves remained identical for at least 50 cycles). All curves displayed in Figure 5 have been obtained in conditions of steady-state response. In all cases, peak currents were directly proportional to the square root of the potential scan rate, indicating that the overall charge transfer processes are diffusion controlled. Indeed, for redox probes immobilized in such insulating mesoporous silica films, their overall electrochemical response is diffusion-controlled, either limited by the pseudo-diffusion of electrons hopping from one redox site to another, or by the diffusion of charge-compensating counter-ions, or a mixture of both processes [46]. No significant difference in the variations of peak potentials with the potential scan rate was observed from one film to another one, suggesting that charge transfer kinetics are not really affected by the amount of [Fe(bpy)_3_]^2+^ species in the films. This appears as an advantage compared to covalently bonded redox moieties in mesoporous silica films (e.g., ferrocene functionalized ones) for which significant charge transfer limitations at low loadings have been reported [46].

### 2.4. Optical Properties of Fe(bpy)_3_^2+^ Modified Sulfonated Silica Films

**Figure 6:**
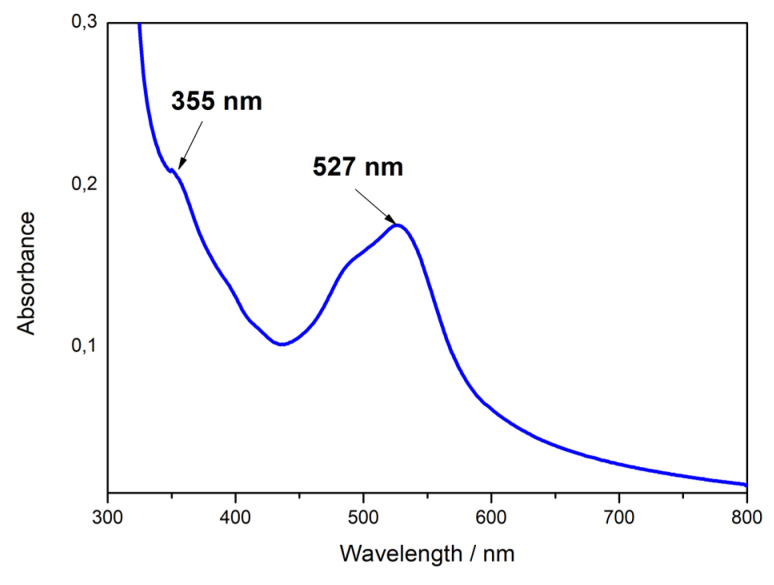
UV–Vis absorption spectrum of a 20/80 MPTMS/TEOS films after immobilizing [Fe(bpy)_3_]^2+^.

Further evidence of the in situ formation of the [Fe(bpy)_3_]^2+^ complex is the coloration change of the Fe^2+^SO_3_^−^@SiO_2_ film when immersed in a 10 mM bipyridine solution in acetonitrile. The optical spectra of Fe(bpy)_3_^2+^ complexes confined in silica films (i.e., the [Fe(bpy)_3_]^2+^SO_3_^−^@SiO_2_ material) were recorded from 300 to 800 nm (Figure 6), absorption measurement below 300 nm is hindered since transmittance of ITO drops sharply in the ultraviolet region due to its narrow optical band gap [47]. The absorption spectrum exhibits a broad band centered at 527 nm with a shoulder on the high energy side, and a second absorption band centered at 355 nm. They can be assigned to metal-to-ligand charge transfer (MLCT) transitions (d(π) → π^*^) [48,49]. These MLCT bands depend on the LUMO energy of polypyridyl ligands. They are very similar to the broad bands centered at 350 and 520 nm, characteristic of Fe(bpy)_3_^2+^ in aqueous solution, and located at absorption wavelength values consistent with those of the literature [50]. The slight shift observed for [Fe(bpy)_3_]^2+^SO_3_^−^@SiO_2_ compared to the bands of solution-phase Fe(bpy)_3_^2+^ complexes is attributed to confinement effects in the silica matrix [51,52]. An additional sharp absorption increase at lower wavelengths is also noticeable and it can be related to the intense absorption band around 300 nm observed for Fe(bpy)_3_^2+^ in solution corresponding to ligand-centered transitions (π → π^*^) of the coordinated bpy ligands [53]. As a control experiment, recording the absorption spectrum for the SO_3_^−^@SiO_2_ films did not show any bands in the visible region, confirming that the bands observed for [Fe(bpy)_3_]^2+^SO_3_^−^@SiO_2_ (Figure 6) are intrinsic to Fe(bpy)_3_^2+^ species confined in the film, and not to the sulfonate functionalized silica film itself. Additionally, the present absorption spectrum for [Fe(bpy)_3_]^2+^SO_3_^−^@SiO_2_ is very similar to the corresponding spectrum previously measured for [Fe(bpy)_3_]^2+^ complexes covalently attached to mesoporous silica thin film [25], suggesting that the way of immobilization does not significantly affect the properties of metal complexes in the confined environment of mesopore channels.

## 3. Materials and Methods

### 3.1. Apparatus

All the electrochemical experiments were performed using a μAutoLab III potentiostat (Eco Chemie) monitored by the GPES software. A three-electrode configuration was employed for all the electrochemical measurements in a one-compartment cell. The voltammetric curves were recorded using a platinum rod as a counter electrode, Ag/AgCl as a reference electrode (Metrohm), and ITO plates (from Delta Technologies; surface resistivity: 8–12 Ω) as working electrodes where the hybrid films were previously deposited. The electrochemically assisted deposition was carried out using a homemade electrochemical cell with a configuration allowing placing the working electrode at the bottom [54]. A stainless-steel counter electrode and a silver wire as a pseudo-reference electrode completed the electrochemical setup.

Several physico-chemical techniques were used to characterize the materials. The mesostructure, morphology, and thickness of the films have been analyzed by transmission electron microscopy (TEM) using an ACCEL ARM 200F microscope at an acceleration voltage of 200 kV. X-ray photoelectron spectroscopy (XPS) characterizations were performed using a KRATOS Axis Ultra X-ray photoelectron spectrometer (Kratos Analytical, Manchester, UK) equipped with a monochromated AlKα X-ray source (hν = 1486.6 eV) operated at 150 W. Raman spectra were recorded using a confocal Raman microscope (inVia^®^ Qontor with a Peltier-cooled CCD camera, Renishaw, Wotton-under-Edge, UK) equipped with a 532 nm laser (irradiance < 5 kW cm^−2^), a 50× objective with a numerical aperture of 0.55, and a 1200 lines.mm^1^ grating. The spectral resolution was about 3 cm^−1^ (10 acquisitions of 4 s). Each sample was analyzed at different points to verify the homogeneity of the studied functionalized silica films. The laser power was optimized to 0.5 mW and the spectrum was recorded in the 50 to 4000 cm^−1^ wavenumber range. The optical absorption spectra were obtained using a Carry 60 UV–Vis spectrophotometer.

### 3.2. Chemicals and Reagents

Tetraethoxysilane (TEOS, 98%, Alfa Aesar), 3-mercaptopropyltrimethoxysilane (MPTMS, 95%, Alfa Aesar), cetyltrimethylammonium bromide (CTAB, 99%, Acros), sodium nitrate (NaNO_3_, 99,5%, Prolabo), iron (II) sulfate heptahydrate (FeSO_4_●7H_2_O, 99%, Aldrich), 2,2’-Bipyridyl (Bpy, 99%, Aldrich), ethanol (95-96%, Merck), hydrochloric acid (37%, Riedel de Haen), and anhydrous acetonitrile (ACN, 99.8%, Aldrich) were used as received without further purification.

### 3.3. Preparation of Thiol and Sulfonate Functionalized Silica Films

Sulfonate-modified silica thin films have been synthesized in two steps involving firstly the synthesis of thiol-modified silica films (MPTMS@SiO_2_) followed by their oxidation using hydrogen peroxide as a chemical oxidizing agent to give the corresponding sulfonate-modified films (SO_3_^−^@SiO_2_). 

The synthesis of MPTMS@SiO_2_ has been achieved by slightly modifying the method reported for non-functionalized films (200 mM TEOS in starting sol) using the EASA method and ITO electrode [20]. The starting sol was constituted in this case of a hydroalcoholic solution (water/ethanol (1:1)) containing 160 mM TEOS, 40 mM MPTMS and CTAB as surfactant template (ratio CTAB/TEOS = 0.32) and 0.1 M NaNO_3_ as supporting electrolyte. The pH of the solution was adjusted to 3 using a 0.1 M HCl aqueous solution. The solution was stirred at room temperature for 2.5 h. Then, a cathodic potential (−1.3 V) was applied for 20 s in a three-electrode system using ITO as a working electrode, in order to deposit the thiol-functionalized vertically oriented film. The electrode was thoroughly rinsed with distilled water and deposited in an oven at 130 °C overnight. The synthesis described above refers to an MPTMS/TEOS concentration ratio of 20%. The same methodology has been used to prepare silica films while varying the MPTMS/TEOS concentration ratio in the starting sol (respectively, 5/95, 10/90, 20/80, and 30/70 MPTMS/TEOS molar ratios were used here). The surfactant (CTAB) was extracted from the mesoporous silica films by immersing them in an ethanol solution containing 0.1 M HCl for 20 min. After surfactant removal, the films were rinsed with distilled water. The sulfonate-functionalized films were obtained by chemical oxidation of the thiol moieties, which was achieved by dipping the thiol-functionalized silica films for 30 min in a solution containing H_2_O_2_ (30%) in 0.1 M H_2_SO_4_ (known to promote the oxidation of thiol groups into sulfonate moieties in mesoporous materials [55]).

### 3.4. Preparation of Fe(bpy)_3_^2+^SO_3_^−^@SiO_2_

The immobilization of [Fe(bpy)_3_]^2+^ complex in the silica nanochannels was done by successive impregnation of sulfonate functionalized mesoporous silica films, respectively, in an aqueous solution of 10 mM FeSO_4_●7H_2_O (for 2h under nitrogen atmosphere in order to avoid the oxidation of the Fe^2+^ ions) to form Fe^2+^SO_3_^−^@SiO_2_ and, after rinsing with acetonitrile, by subsequent immersion in a solution containing 30 mM bipyridine in acetonitrile and let reacting for 30 min to generate Fe(bpy)_3_^2+^SO_3_^−^@SiO_2_. A final washing with acetonitrile completed the process.

## 4. Conclusions

In the present work, we have demonstrated that organosulfonate groups grafted to the internal walls of mesoporous silica films can be used as second sphere coordination ligands in the case of first-row transition metal ions that can be used afterwards to form in situ coordination complexes in one step, as demonstrated here for Fe(bpy)_3_^2+^ species. Their structural, optical, and electrochemical properties are preserved when immobilized in the mesoporous silica matrix. A huge amount of Fe(bpy)_3_^2+^ species can be confined in the films, up to 18.6 × 10^−10^ mol for a film prepared from a sol medium with an MPTMS/TEOS ratio of 20/80. The proposed approach could be extended to the confinement of other metal polypyridyl complexes, individually or in a mixture. Such optically and electrochemically active complexes confined in transparent thin films look promising for the development of optical, optoelectronic, or photoswitchable devices. 

## Figures and Tables

**Table 1:** Atomic composition (% At.) and binding energies (BE) for the functionalized mesoporous silica films at each step of the modification process (data obtained from the XPS measurements).

Films Composition	Si2p Signal		S2p Signal		Fe2p Signal		N1s Signal		Atomic Ratios		
	%At	BE/Ev	%At	BE/Ev	%At	BE/Ev	%At	BE/Ev	S/Si	S/Fe	N/Fe
MCTMS/TEOS											
5/95	95.8	102.0	4.2	161.7	- - - -	- - - -	- - - -	- - - -	0.04	- - - -	- - - -
10/90	90.5	102.0	9.5	161.7	- - - -	- - - -	- - - -	- - - -	0.11	- - - -	- - - -
20/80	81.2	102.0	18.8	161.8	- - - -	- - - -	- - - -	- - - -	0.23	- - - -	- - - -
30/70	72.3	102.0	27.7	161.7	- - - -	- - - -	- - - -	- - - -	0.38	- - - -	- - - -
SO_3_^−^/TEOS											
5/95	96.0	102.0	4.0	167.1	- - - -	- - - -	- - - -	- - - -	0.04	- - - -	- - - -
10/90	96.7	102.0	9.3	167.2	- - - -	- - - -	- - - -	- - - -	0.10	- - - -	- - - -
20/80	81.6	102.0	18.4	167.2	- - - -	- - - -	- - - -	- - - -	0.23	- - - -	- - - -
30/70	71.5	102.0	28.5	167.2	- - - -	- - - -	- - - -	- - - -	0.40	- - - -	- - - -
Fe^2+^SO_3_^−^/TEOS											
5/95	94.3	102.0	3.8	167.7	1.9	710.7/723.4	- - - -	- - - -	0.04	2.00	- - - -
10/90	88.1	102.0	8.5	167.7	3.7	710.8/723.5	- - - -	- - - -	0.10	2.30	- - - -
20/80	79.9	102.0	14.2	167.8	5.9	710.7/723.4	- - - -	- - - -	0.18	2.41	- - - -
30/70	69.9	102.0	23.1	167.8	7.0	710.7/723.4	- - - -	- - - -	0.33	3.30	- - - -
[Fe(bpy)_3_]^2+^ SO_3_^−^/TEOS											
5/95	87.5	102.0	2.9	167.5	1.4	710.7/723.4	8.2	399.9	0.03	2.10	5.86
10/90	74.7	102.0	5.9	167.4	2.9	710.8/723.4	16.5	399.8	0.08	2.03	5.69
20/80	48.5	102.0	11.1	167.4	5.5	710.9/723.3	31.9	399.7	0.23	2.02	5.80
30/70	65.3	102.0	22.5	167.4	6.1	710.9/723.4	6.1	399.8	0.34	3.69	1.00

**Table 2:** Identified Raman vibration modes and assignment for [Fe(bpy)_3_]^2+^SO_3_^−^@SiO_2_.

Peak Position (cm^−1^)	Assignment
155	δ (N-Fe-N); benzene cycle bending modes
369	ν (Fe-N)
662	Ring deformation
766	Ring deformation
1022	Ring deformation
1275	δ (C-H)
1322	δ (C-H)
1489	ν (C=C)
1562	δ (C-H)
1605	ν (C=N)

## Data Availability

Not applicable.

## Supplementary Materials

The following supporting information can be downloaded at: https://www.mdpi.com/article/10.3390/molecules27175444/s1, Figure S1: Cyclic voltammograms recorded in aqueous 0.1M NaNO_3_ solution after impregnation of a silica film in 10 mM FeCl_3_●6H_2_O solution for 2 h. The silica film was prepared by EASA method from a solution containing 200 mM of hydrolyzed tetraethoxysilane, 64 mM CTAB and 0.1 M NaNO_3_.; Figure S2: XPS survey spectra obtained for various functionalized silica films on ITO, respectively, (a) [Fe(bpy)_3_]^2+^SO_3_^−^@SiO_2_, (b) Fe^2+^SO_3_^−^@SiO_2_, (c) SO_3_^−^@SiO_2_, and (d) SH@SiO_2_.
