# Supplementary material for: Weak Coordinating Character of Organosulfonates in Oriented Silica Films: An Efficient Approach for Immobilizing Cationic Metal-Transition Complexes
Source: Molecules. 2022 Aug 25;27(17):5444. doi: 10.3390/molecules27175444 (PMC9458166; doi:10.3390/molecules27175444)
Supplement: Supplementary file 1 [file molecules-27-05444-s001.zip › molecules-1851622-supplementary.pdf]

Supporting Information

Non-innocent weak coordinating character of organosulfonates assisted by hydrogen bonding in oriented silica films: an efficient approach for immobilizing cationic metal-transition complexes

Samuel Ahoulou[1,2], Clara Richart,[1] Cédric Carteret,[1] Sébastien Pillet,[2*] Neus Vilà[1*] and Alain Walcarius[1*]

1 *Université de Lorraine, CNRS, LCPME, Nancy, France*
2 *Université de Lorraine, CNRS, CRM2, Nancy, France*
* Correspondence: neus.vila@univ-lorraine.fr; sebastien.pillet@univ-lorraine.fr; alain.walcarius@univ-lorraine.fr;

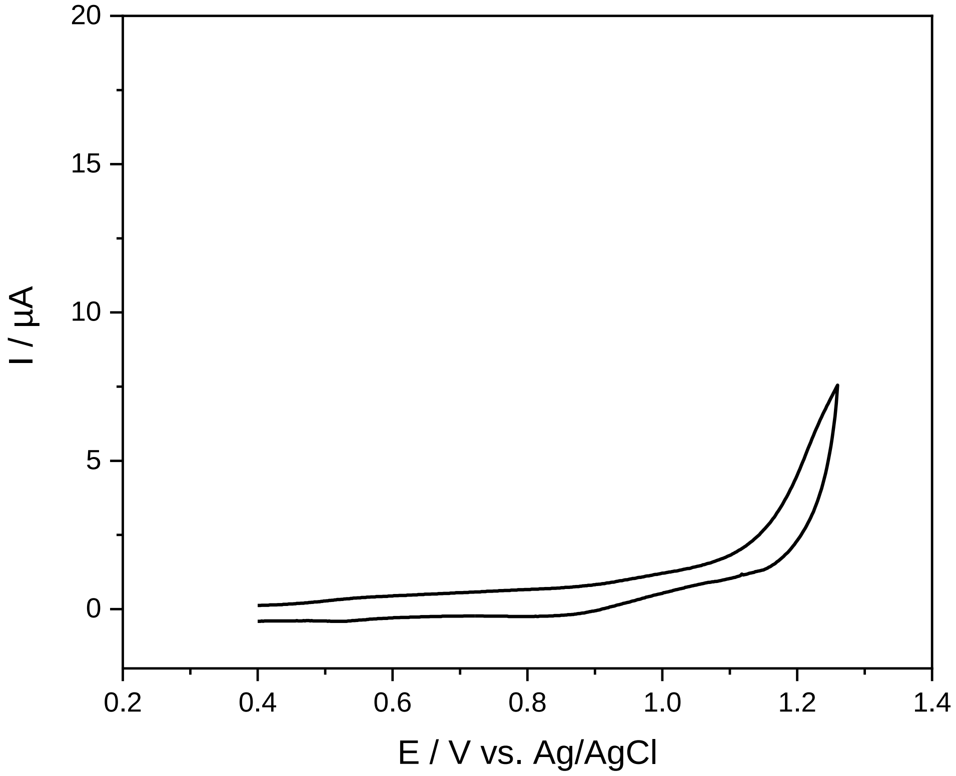

Figure S1. Cyclic voltammograms recorded in aqueous 0.1M $NaNO_3$ solution after impregnation of a silica film in 10 mM $FeCl_3 \bullet 6H_2O$ solution for 2h. The silica film was prepared by EASA method from a solution containing 200 mM of hydrolyzed tetraethoxysilane, 64 mM CTAB and 0.1 M $NaNO_3$.

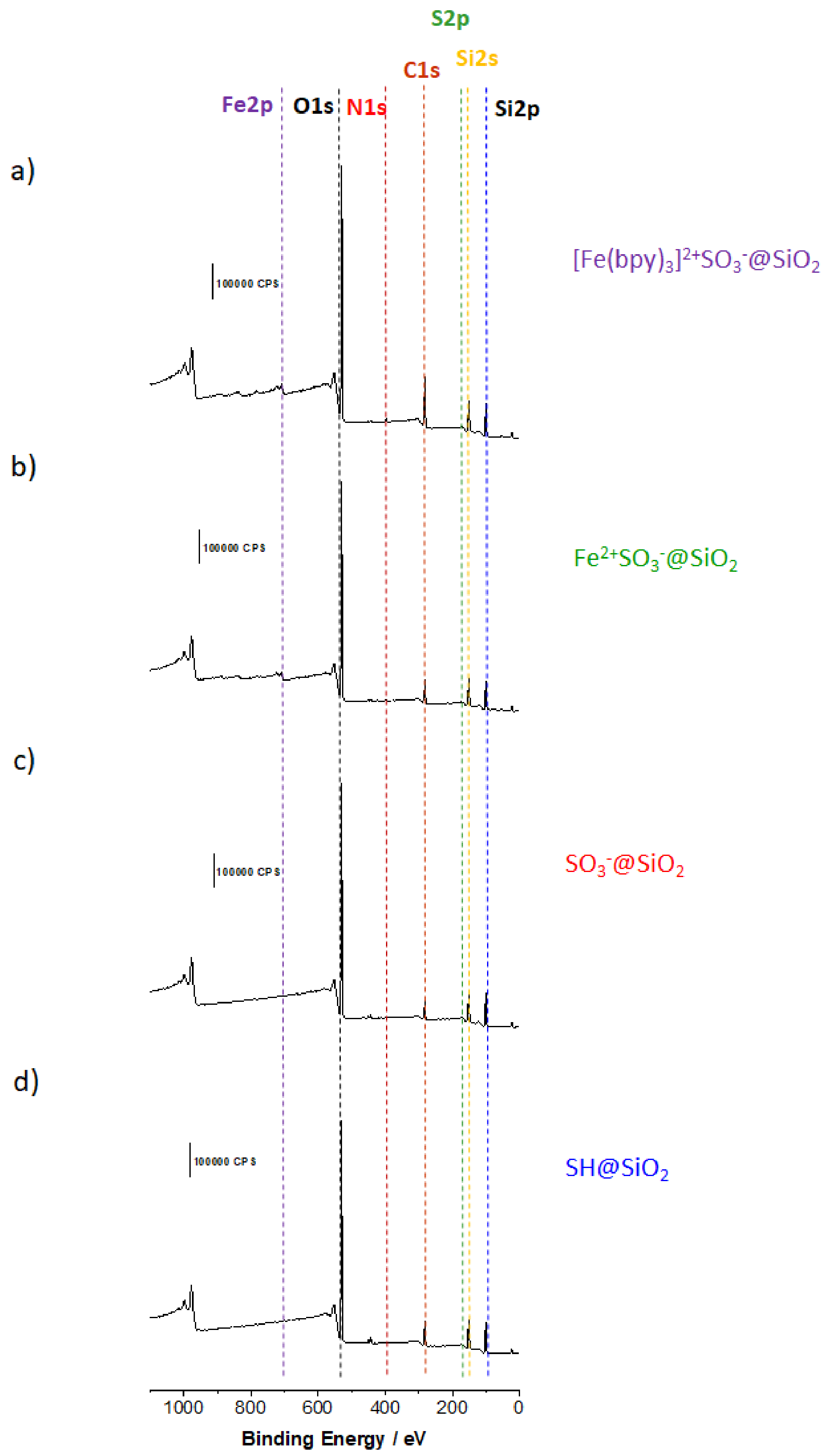

Figure S2. XPS survey spectra obtained for various functionalized silica films on ITO, respectively (a) $[Fe(bpy)_3]^{2+}SO_3^-@SiO_2$, (b) $Fe^{2+}SO_3^-@SiO_2$, (c) $SO_3^-@SiO_2$, and (d) $SH@SiO_2$.
